# Automating Quality Metrics in the Era of Electronic Medical Records: Digital Signatures for Ventilator Bundle Compliance

**DOI:** 10.1155/2015/396508

**Published:** 2015-06-08

**Authors:** Haitao Lan, Charat Thongprayoon, Adil Ahmed, Vitaly Herasevich, Priya Sampathkumar, Ognjen Gajic, John C. O'Horo

**Affiliations:** ^1^Guang'anmen Hospital, China Academy of Chinese Medicine Sciences, Beijing 100053, China; ^2^Multidisciplinary Epidemiology and Translational Research in Critical Care, Perioperative and Emergency Medicine (METRIC-PM) Group, USA; ^3^Mayo Clinic, Department of Medicine, Division of Pulmonary and Critical Care Medicine, Rochester, MN 55905, USA; ^4^North Central Texas Medical Foundation, Wichita Falls Family Practice Residency Program, Wichita Falls, TX 76301, USA; ^5^Mayo Clinic, Department of Anesthesiology, Division Critical Care Medicine, Rochester, MN 55905, USA; ^6^Mayo Clinic, Department of Medicine, Division of Infectious Diseases, Rochester, MN 55905, USA

## Abstract

Ventilator-associated events (VAEs) are associated with increased risk of poor outcomes, including death. Bundle practices including thromboembolism prophylaxis, stress ulcer prophylaxis, oral care, and daily sedation breaks and spontaneous breathing trials aim to reduce rates of VAEs and are endorsed as quality metrics in the intensive care units. We sought to create electronic search algorithms (digital signatures) to evaluate compliance with ventilator bundle components as the first step in a larger project evaluating the ventilator bundle effect on VAE. We developed digital signatures of bundle compliance using a retrospective cohort of 542 ICU patients from 2010 for derivation and validation and testing of signature accuracy from a cohort of random 100 patients from 2012. Accuracy was evaluated against manual chart review. Overall, digital signatures performed well, with median sensitivity of 100% (range, 94.4%–100%) and median specificity of 100% (range, 100%–99.8%). Automated ascertainment from electronic medical records accurately assesses ventilator bundle compliance and can be used for quality reporting and research in VAE.

## 1. Introduction

Patients who receive mechanical ventilation are at high risk of complications and poor outcomes including death [1]. To effectively manage these high risk patients, providers are encouraged to put in place best practice “bundles” addressing the use of deep vein thrombosis (DVT) prophylaxis, peptic ulcer prophylaxis, oral hygiene, elevation of the head of the bed, daily sedation holiday, and daily spontaneous breathing trial [2]. The ventilator bundle has formed the backbone of many quality improvement efforts and metrics for intensive care units, though its impact on patient outcomes remains uncertain [3]. In 2011, CDC/NHSN proposed a new approach to surveillance including a broader range of ventilator complications termed ventilator-associated events (VAEs) [4]. We sought to investigate if compliance with ventilator bundle practices effectively reduces the risk of the broader set of VAEs and evaluate the relative contribution of each bundle element to patient outcomes. In order to accomplish this, we needed to develop a reliable strategy for assessing bundle compliance for a large number of patients in an efficient manner.

Manual chart review is the “gold standard” of retrospective studies. However, it is time-consuming, inaccurate, resource intense, and not feasible for large sample sizes. The recent development of information technology and the widespread use of electronic medical record (EMR) systems [5] make it possible to develop electronic search algorithms (hereafter referred to as digital signatures) to automatically search patient charts quickly and efficiently. The digital signature also can be translated into a real time automated algorithms or “sniffers,” where the same rules that were used to retrospectively search charts electronically can give real time or near real time reports and alerts to improve patient care [6].

This study aimed to develop and validate digital signatures for each part of the ventilator bundle, including DVT prophylaxis, peptic ulcer prophylaxis, oral care, head of bed elevation, and sedation breaks.

## 2. Materials and Methods

We designed this study as a retrospective study with both derivation and validation cohorts ascertained from intensive care unit patients. The Mayo Clinic Institutional Review Board approved the study as minimal risk research with waived informed consent.

### 2.1. Study Population

We used a retrospective cohort of 1000 randomly selected patients who were admitted to the intensive care unit (ICU) for at least two consecutive days during 2010 to form our derivation cohort. Of these, 542 met our study inclusion criteria including two consecutive days of mechanical ventilation and research authorization. Our derivation cohort included both ventilated and nonventilated patients to ensure we would have an adequate number of both “true positive” and “true negative” compliance for each element of the bundle while adjusting our search strategy. We then validated the electronic data extraction strategy in an independent cohort of 100 randomly selected patients who were mechanically ventilated for at least two consecutive days in 2012. The purpose of the selection of mechanically ventilated patients from two different years was to better assess the performance of the strategy. Patients aged < 18 year or without research authorization were excluded.

### 2.2. Electronic Data Extraction

To develop the electronic data extraction strategy, we utilized data from a custom integrative relational research database that contains a near real-time copy of clinical and administrative data from the electronic medical record (EMR). The Multidisciplinary Epidemiology and Translational Research in Intensive Care (METRIC) datamart accumulates pertinent vital signs, fluid input/output, and medication administration record data within an average of 15 minutes from its entry into the EMR and serves as the main data repository for data rule development. More detailed structures and contents have been previously published [7].

**Figure 1:**
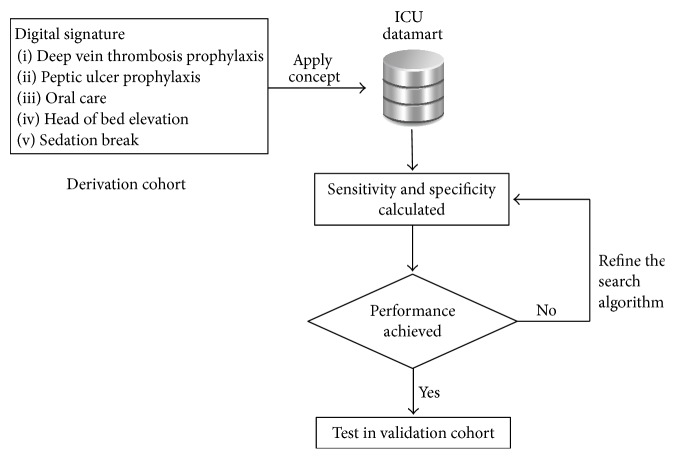
Flow chart for digital signature derivation and validation. This model was applied to all signatures developed in the present study.

For each bundle element, we iteratively improved the accuracy of our electronic query using the derivation cohort (Figure 1: flow chart). In all iterations, we calculated and analyzed sensitivity and specificity compared to the reference standard and examined discordant pairs for data which could be used to improve the electronic search accuracy. Once we achieved acceptable sensitivity and specificity, we validated our queries in another independent cohort and calculated final sensitivity and specificity of our digital signatures. The final electronic queries for each ventilator compliance bundle were presented in Table 1.

### 2.3. Reference Standard

The reference standard was defined as the agreement between manual and electronic data extraction. A trained investigator (LH), who was blinded to electronic data extraction result, performed comprehensive medical record review to identify the presence or absence of each component of ventilator compliance bundle according to predefined definition (Table 1) between 00:00 to 23:59 on ICU day 2 in the derivation cohort and mechanical ventilator day 2 in the validation cohort. In case there was a disagreement between manual and electronic data extraction, a third independent investigator (JCO), who was blinded to both results, would make the final adjudication; this definition has been previously used [8].

### 2.4. Statistical Analyses

We summarized clinical characteristics of derivation and validation cohorts using mean ± SD for continuous variables and using counts with percentages for categorical variables. We calculated sensitivity and specificity of each electronic data extraction based on the comparison of the test result and reference standard in the two cohorts. The 95% confidence intervals were calculated using an exact test for proportions. JMP statistical software (version 9.0, SAS Institute Inc.©) was used for all data analysis and randomization.

## 3. Results and Discussion

The derivation subset included a total of 542 ICU patients randomly selected from January 2010 to December 2010. The validation subset included a total of 100 randomly selected patients from January 2012 to December 2012. There were no differences in age, gender, and race between the two groups. The demographic characteristics and baseline of the derivation and validation subset are summarized in Table 2.

The sensitivities of five ventilator bundle components were from 92% to 100% in the derivation subset in our final iteration. The specificities ranged from 50% to 99.8% after modification. Elevation of the head of the bed was the bundle element that could not be improved to an adequate sensitivity or specificity because of variable and inconsistent charting. We thus decided not to validate this query and did not test in our validation cohort. When examining the validation cohort, the sensitivities of our digital signatures ranged from 94.4% to 100%, and specificity was 100% for each (Table 3).

Manual chart review was slow, requiring our reviewers to access two or more programs to abstract the relevant data from the EMR, taking an average of 10 minutes/patient. We achieved comparable results with electronic data abstraction, which will allow us to scan compliance of thousands of patients in a reasonable time frame for the second part of our study, an assessment of ventilator bundle compliance on the risk of developing a VAE.

With the widespread adoption of EMRs, the digital signature is an increasingly attractive alternative to manual chart review. Digital signatures have several advantages. First, they are more efficient, making larger-scale cohort studies practical without significant personnel or time expenditure. Second, in developing them, we can look for markers of specific activities that correlate with actual patient outcomes and thus mitigate some types of reporting bias. For example, our DVT prophylaxis signature looks for times where one of the commonly used agents is actually administered, as opposed to asking staff to fill out a checkbox saying that “DVT prophylaxis has been addressed.” More broadly, this kind of search allows automated searching beyond simple billing codes and administrative data, which are notoriously variable in accuracy [9–12].

Finally, digital signatures have the potential to be translated into real-time electronic search algorithms, or “sniffers,” to provide near real time data. For example, the same rules that we used to develop our peptic ulcer prophylaxis signature could provide real time data on compliance, use and misuse. Sniffers are increasingly prevalent, though a recent systematic review highlighted issues with variable performance and accuracy owing in part to inadequate validation [13]. As we noted in our effort to derive and validate a signature for head of bed elevation, variability in documentation practice may limit the ability to derive a clinically useful digital signature; however, an emerging automatic documentation technology could help overcome this limitation.

An interesting feature we noted in our validation cohort was higher diagnostic performance than in our derivation cohort. As our derivation cohort was what we used to derive the search, we expected to be “overfitted” to that set and lose both sensitivity and specificity as we moved to another cohort. However, we instead noted improvement. This probably owes to improvements made in the ICU datamart's accuracy over time, as our derivation cohort was from archived data in 2010, and validation used the same rules in 2012. We noted better agreement between datamart and EMR data in the more recent set and thus better improvement with our rules-based signatures.

With reasonable search algorithms, this allows us to move forward and evaluate the efficacy of specific ventilator bundle elements in preventing VAE. A previous study at our institution using pre- and postbundle implementation measures found no effect, but that study was an ecological design and was not able to evaluate individual patient bundle compliance [3]. With these signatures, we will be able to give a higher resolution evaluation of the effect of the ventilator bundle. We can also work towards developing real time compliance monitoring of the ventilator bundle for both quality improvement purposes, aiming to indirectly improve care and reduce costs with passive monitoring of value-adding practices.

Our study also has several limitations. First, as noted above, we are limited by what is electronically documented and the accuracy of initial inputs. Second, preferred medications and formularies differ between hospitals, and while our digital signature may be a starting point for other hospitals attempting something similar, calibration and validation would be necessary to generalize this elsewhere. Finally, the single-center, academic nature of our institution could raise the concern of referral bias and further limit generalizability of our approach.

## 4. Conclusion

The digital signatures used to extract and screen the usage of ventilator-associated pneumonia bundle elements were both sensitive and specific for DVT prophylaxis, peptic ulcer prophylaxis, daily sedation break, and oral care. We were not able to derive a similarly useful signature for head of bed elevation. These signatures have acceptable sensitivity and specificity for use in our larger study of the impact of the ventilator bundle on risk of VAE.

## Figures and Tables

**Table 1:** Bundle components and definitions. The “medical definition” refers to the objective of the bundle element. The “EMR definition” is how we operationalized this for our digital signature. The EMR section used refers to portions of the patient chart searched with the digital signature for the bundle element.

Ventilator compliance bundle element	Medical definition	EMR definition	EMR section used
DVT prophylaxis	The presence of an appropriate anticoagulant within 24-hour period	The systemic administration of one of the following medications within 24 hours regardless of dosage use: (i) Argatroban (ii) Bivalirudin (iii) Dabigatran (iv) Dalteparin (v) Enoxaparin (vi) Fondaparinux (vii) Heparin^*∗*^ (viii) Lepirudin (ix) Rivaroxaban (x) Warfarin	Medication administration record, fluid data

Peptic ulcer prophylaxis	The presence of an appropriate acid-inhibitory drug or sucralfate within 24-hour period	The systemic administration of one of the following medications within 24 hours regardless of dosage use: (i) Cimetidine (ii) Dexlansoprazole (iii) Esomeprazole (iv) Famotidine (v) Lansoprazole (vi) Nizatidine (vii) Omeprazole (viii) Pantoprazole (ix) Rabeprazole (x) Ranitidine (xi) Sucralfate	Medication administration record, fluid data

Oral care	The presence of chlorhexidine oral care within 24-hour period	The use of chlorhexidine oral rinse within 24 hours	Medication administration record

Head of bed elevation	≥30 degree consistently documented within 24-hour period	The patient position was one of the following: (i) Head of bed ≥ 30 degrees (ii) Chair (iii) Wheelchair (iv) Dangle (v) Semifowler (vi) Upright	Nursing flow sheet

Sedation break	If continuous IV sedation is present, any continuous intravenous sedatives or opioids break for ≥ 15 minutes was performed within 24 hour period	(1) Identify the continuous IV administration of one of the following medications within 24 hours regardless of duration and dosage use: (i) Lorazepam (ii) Fentanyl (iii) Hydromorphone (iv) Midazolam (v) Morphine (vi) Propofol (2) Identify sedation break action when IV infusion dose = 0 or task status description = “disconnect” (3) Identify actual sedation break when there is no infusion of the same sedation within 15 minutes after sedation break occurs	Fluid data

^*∗*^Excepting heparin locks and line flushes.

**Table 2:** Clinical characteristics of derivation and validation cohort.

Variable	Derivation cohort^*∗*^	Validation cohort^*∗*^	*P* value^*∗∗*^
*N*	542	100	
Age (year)	63 ± 17	62 ± 17	0.59
Male sex	311 (57)	61 (61)	0.51
White	477 (88)	90 (90)	0.73
Medical ICU	207 (38)	51 (51)	<0.01
Admission SOFA score	8 ± 3	8 ± 4	0.99
Admission APACHE score	76 ± 25	85 ± 27	<0.01
MV use on reviewed day^#^	352 (65)	100 (100)	<0.01
ICU length of stay (day)	2.4 (1.5–5.3)	5.8 (2.8–10.7)	<0.01
ICU mortality	34 (6)	7 (7)	0.82
Hospital mortality	54 (10)	14 (14)	0.22

^*∗*^Continuous data are presented as mean ± SD if normally distributed, median (25th percentile–75th percentile) for nonnormal data; categorical variables are reported as count (%).

^#^ICU day 2 for derivation cohort and mechanical ventilator day 2 for validation cohort.

^*∗∗*^ *P* value calculated by Fisher Chi-Square for categorical variables, Student's *t*-test for normally distributed continuous variables, and Wilcoxon rank-sum for nonparametric analysis.

**Table 3:** Sensitivity, specificity, and the concordance and discordance between electronic data extraction result and reference standard in derivation and validation cohort.

Item	Cohort	Number of patients	Sensitivity (95% CI)	Specificity (95% CI)	TP	TN	FP	FN
DVT prophylaxis	DC	542	91.7 (87.7–94.7)	93.5 (89.9–96.1)	243	259	18	22
	VC	100	100 (92.8, 100)	100 (92.8, 100)	50	50	0	0

Peptic ulcer prophylaxis	DC	542	94.1 (91.0–96.3)	96.6 (93.1–98.6)	317	198	7	20
	VC	100	100 (95.9, 100)	100 (95.9, 100)	89	11	0	0

Oral care	DC	542	100 (96.7–100)	99.8 (98.7–100)	111	430	1	0
	VC	100	100 (95, 100)	100 (87, 100)	73	27	0	0

Head of bed elevation	DC	542	96.5 (95.5–97.9)	50 (32.4–67.6)	490	17	17	18
	VC	N/A^*∗*^	N/A^*∗*^	N/A^*∗*^	N/A^*∗*^	N/A^*∗*^	N/A^*∗*^	N/A^*∗*^

Sedation break	DC	254^*∗∗*^	100 (98.3–100)	87.9 (71.8–96.5)	221	29	4	0
	VC	73^*∗∗*^	94.4 (84.6, 98.8)	100 (83, 100)	51	20	0	3

CI: confidence interval; DC: derivation cohort; VC: validation cohort; DVT: deep vein thrombosis; TP: true positive; FP: false positive; TN: true negative; FN: false negative.

^*∗*^Because we could never achieve high specificity with the head of bed elevation in the derivation cohort, we did not attempt validation.

^*∗∗*^Only patients on sedation at any point during the test day were assessed for “sedation break.”
